# CAR T cell therapy for refractory pediatric systemic lupus erythematosus: a new era of hope?

**DOI:** 10.1186/s12969-024-00990-4

**Published:** 2024-08-08

**Authors:** Ivana Stojkic, Lauren Harper, Samantha Coss, Mahmoud Kallash, Kyla Driest, Margaret Lamb, Stacy P. Ardoin, Shoghik Akoghlanian

**Affiliations:** 1 Division of Pediatric Rheumatology, Nationwide Children’s Hospital, Columbus, OH 46205 USA; 2 Division of Hematology and Oncology, Nationwide Children’s Hospital, Columbus, OH USA; 3 Division of Nephrology, Nationwide Children’s Hospital, Columbus, OH USA

**Keywords:** SLE, CAR T, B cells

## Abstract

Systemic lupus erythematosus (SLE) is a chronic autoimmune condition that can affect multiple organ systems and is heterogenous in its presentation and response to therapy. When diagnosed in childhood, SLE is associated with increased morbidity and mortality compared to adult SLE, often requiring substantial immunosuppression with the risk of significant side effects. There remains a significant unmet need for new therapies that can improve disease control and reduce glucocorticoid and other toxic medication exposure for patients with severe or refractory disease. The pathogenesis of SLE involves B cell dysregulation and autoantibody production, which are a hallmark of the disease. Currently approved B cell directed therapies often result in incomplete B cell depletion and may not target long-lived plasma cells responsible for SLE autoantibodies. It is hypothesized that by persistently eliminating both B cells and plasmablasts, CAR T therapy can halt autoimmunity and prevent organ damage in patient’s refractory to current B cell-depleting treatments. Herein we summarize the current preclinical and clinical data utilizing CAR T cells for SLE and discuss the future of this treatment modality for lupus.

**Supplementary Information:**

The online version contains supplementary material available at 10.1186/s12969-024-00990-4.

## Background

Systemic lupus erythematosus (SLE) is a chronic autoimmune condition that can affect multiple organ systems and is heterogenous in its presentation and response to therapy. SLE remains incurable and has significant negative impacts on the function and quality of life of affected individuals. While the current therapies used to treat SLE have led to an improvement in morbidity and mortality, many patients with SLE remain refractory to available treatment regimens [1, 2]. Additionally, when diagnosed in childhood, SLE is associated with increased morbidity and mortality compared to adult SLE [3, 4]. SLE is known to be more aggressive in non-white ethnicities [5]. Lupus nephritis (LN) in particular carries significant morbidity and has an increased prevalence and severity in children and adolescents compared to adults [6, 7].

Despite advances in therapy [8, 9], the survival rates for SLE and LN have plateaued over recent decades [10]. In the United States, SLE is the tenth leading cause of death and the number one cause of death from a chronic inflammatory disease for females in the 15–24 year age group [11]. Complete remission of LN is achieved in only 40–60% of youth despite aggressive therapy [12]. Children who develop end stage kidney disease (ESKD) due to LN have a 5-year mortality of approximately 22% [13]. Compared to other children on hemodialysis, those with LN have an increased mortality rate and are less likely to receive renal transplant despite the fact that post-transplant, graft survival and infection rates are comparable [14].

Due to increased disease severity, most children with SLE require prolonged treatment with high dose glucocorticoids and aggressive immunosuppression often with cyclcophosphamide (CYC). They can have high cumulative exposure to these therapies that carry significant morbidity and mortality, particularly from infection [15]. Glucocorticoids carry a significant morbidity risk in SLE in particular with increased risk of lupus-related damage as well as adverse effects like cataracts, hypertension, hyperglycemia, dyslipidemia, avascular necrosis, obesity and poor growth, and osteoporosis [16]. Therefore, there remains a significant unmet need for new therapies that can improve disease control and reduce glucocorticoid and other toxic medication exposure for these patients.

### Disease pathogenesis and targeted treatments

Both the innate and adaptive immune responses are implicated in the pathogenesis of SLE [17–19]. The innate immune system includes complement proteins, macrophages, neutrophils, antigen presenting cells, and various antimicrobial molecules and support cells. In SLE, there is poor clearance of apoptotic material allowing for the presentation of intracellular substances that then trigger the inflammatory cascade [20–23]. Abnormalities and defects in the complement pathway [24] and the Fas ligand pathway [25–27] are also involved in its pathogenesis. Defects in neutrophil apoptosis result in increased antigen production and triggering of inflammation [28]. The adaptive immune system includes T cells, B cells, and natural killer cells. In SLE, B cells are dysregulated and produce autoantibodies, which are a hallmark of SLE [29–31]. Autoantibodies are thought to contribute to SLE pathogenesis in many ways, including from deposition as well as direct damage to tissues, stimulation of interferon production and signaling, and binding to and increasing the immunogenicity of neutrophil extracellular traps [32, 33].

Despite a multimodal approach to target the different aspects of the immune system involved in SLE pathogenesis, many patients have refractory disease, defined as a “failure to improve within 3–4 months or the inability to achieve partial remission after 5–12 months or complete remission after 2 years of treatment” [34, 35]. In most studies, SLE is also considered refractory when patients do not respond to 1–3 immunosuppressive medications in addition to corticosteroids [35]. Given that so many therapeutic options are exhausted in these patients, they are often reliant on glucocorticoids for disease control.

Widespread implementation of treatments such as CYC has improved outcomes for patients with refractory disease, but gains have been more modest in patients with severe kidney disease [10, 36].

### B cell-directed therapies

Recent research has turned to B cell therapies to try and improve outcomes in SLE and LN [37, 38]. B cells produce autoantibodies, which are known to play a critical role in SLE pathogenesis [39, 40] In addition, B lymphocytes indirectly affect antigen-presenting activity in SLE [41]. Rituximab, a monoclonal antibody that targets CD20-expressing B cells, is used to treat lupus with mixed effects [42]. In the LN Assessment with Rituximab (LUNAR) trial for adults with SLE, rituximab was not shown to be effective in reaching the endpoints of improved clinical renal outcomes after 1 year of therapy [43]. The Exploratory Phase II/III SLE Evaluation of Rituximab (EXPLORER) trial sought to evaluate if there was a major or partial clinical response for moderate to severe extrarenal manifestation of adult SLE [44]. Likewise, it did not reach its end goal. Despite these studies not reaching their endpoints, rituximab has continued to be used clinically for the treatment of lupus due to real-world experience supporting its efficacy. Trial design may have contributed to the LUNAR and EXPLORER studies not meeting their primary endpoints– including poorly targeted patient selection, inclusion of patients on excessively high doses of corticosteroids, and inadequate study time to reach ambitious endpoints [35]. In a meta-analysis that included 31 studies involving over a thousand SLE patients, the global response to rituximab was 72% with a complete response in 46% and a partial response rate of 32% [41].

Belimumab is a fully humanized monoclonal antibody against B lymphocyte stimulator (BAFF), an essential survival factor for B cells, that is used as an adjunct B cells depleting agent for lupus treatment. Two international phase III clinical trials, BLISS-52 [45] and BLISS-76 [46], of adults with SLE led to the U.S. Food and Drug Administration FDA approval of belimumab for lupus in 2011, the first new treatment for lupus in over 50 years. Subanalysis of those studies suggested its benefit in LN. BLISS-LN was conducted and randomized 448 adult SLE patients with active, biopsy-proven class III, IV and/or V LN 1:1 to receive belimumab or placebo, plus standard of care therapy, for 104 weeks. The primary endpoint was met by 43% of patients receiving belimumab compared to 32% on placebo. A pediatric trial of belimumab, PLUTO [47], showed similar efficacy compared to the adult data with 41.2% achieving SRI response rate [48]. Thus, numerous studies have shown that anti-B cell therapies may serve as a potent treatment for SLE [49], even though we do not always see an adequate response in every patient. It has been postulated that the failure of current B cell therapies is largely related to transient or incomplete depletion of B cells, as has been seen in both rheumatoid arthritis and in SLE [50, 51].

## Chimeric antigen receptor T Cells

Chimeric antigen receptor T cells (CAR T) are T cells that are genetically engineered to express a CAR to target specific surface antigens without the need for MHC presentation. A CAR combines an antibody derived scFV extracellular domain with an intracellular domain designed to stimulate T cell activation. The most common clinically utilized CAR T cells have an intracellular domain composed of a CD3𝛇 stimulatory domain combined with either 4-1BB or CD28 costimulatory domains. CAR T cells were first developed in the 1980s, however, the major clinical breakthrough occurred over 20 years later with the first human trials of anti-CD19 CAR T cells for B cell malignancies [52]. Tisagenlecleucel, an autologous anti-CD19 CAR T cell product, was the first CAR T cell product utilized in pediatric cancer. In phase I/II clinical trials of tisagenlecleucel in pediatric patients with relapsed/refractory acute lymphoblastic leukemia, complete response rates were > 80% after a single dose [53]. These dramatic response rates led to FDA approval of tisagenlecleucel in 2017, and since then 5 other CAR T cell products have been approved for adults with B cell lymphomas and multiple myeloma. The majority of CAR T cell studies utilize autologous T cells collected via apheresis. CAR T cell manufacturing times vary but may take up to 2 weeks for CAR transduction and T cell expansion. Prior to infusion, patients undergo lymphodepleting chemotherapy to enhance CAR T cell in vivo expansion and persistence. Typical regimens for lymphodepletion include both CYC and fludarabine.

As CD19 CAR T cells can deplete both normal and pathogenic B cells, as well as plasmablasts, there is promise in utilizing this novel therapeutic avenue for SLE [52]. It is hypothesized that by potently eliminating both B cells and plasmablasts, CAR T therapy can halt autoimmunity and prevent organ damage in patients refractory to current B cell-depleting treatments.

To that end, CD19 CAR T cells have demonstrated efficacy in proof-of-concept murine models of lupus [54, 55]. MRL-lpr is a spontaneous SLE murine model with severe lupus nephritis. Infusion of syngeneic anti-mouse CD19 CAR T cells into 13-week-old MRL-lpr mice after low dose irradiation resulted in nearly complete eradication of circulating CD19 + B cells in the blood, which correlated with improved survival and less active disease [54]. Importantly, the mice spared total body irradiation prior to the CD19 CAR T infusion did not exhibit such robust B cell depletion. Similarly, it was demonstrated that infusion of anti-CD19 CD8 + T cells into MRL-lpr and NZB/W mice led to profound and prolonged depletion of CD19 + B cells with aplasia lasting more than one year post therapy [55]. Anti-DNA IgG and IgM antibodies that were detectable before CAR T cell injections declined to undetectable levels after treatment and remained low to undetectable in most of the CAR T cell-treated mice for at least 19 weeks post infusion. These results correlated to a drastic improvement in survival and reversal of high-grade proteinuria in the CAR T treated mice [55].

## CAR T cells in human SLE

Given this promising pre-clinical data and substantial clinical experience with CD19 CAR T cells for hematologic malignancies, there is a large interest in utilizing CD19 CAR T cells for autoimmune disease. Early clinical success has been demonstrated in patients with SLE, anti-synthetase syndrome, and myasthenia gravis [56–58]. In 2023, Schett and colleagues, published a comprehensive review about CAR T therapy in autoimmune disease including lupus. The first report of CAR T to treat human SLE was published by a group in Germany in 2021. Mougiakakos, D et al., reported a case of a 20-year-old woman with severe and refractory SLE who presented with active LN (class IIIA), nephrotic syndrome, pericarditis, pleurisy, rash, arthritis, and a history of Libman–Sacks endocarditis whose lupus was inadequately controlled despite treatment with hydroxychloroquine, high-dose glucocorticoids, CYC, mycophenolate mofetil (MMF), tacrolimus, belimumab, and rituximab [59]. After lymphodepletion with fludarabine and CYC, a single dose of 1.1 × 10^6^ CD19 CAR T cells/kg (CD4 + to CD8 + T cell ratio of 3:1) was administered. The common side effects of CAR T therapy including cytokine release syndrome (CRS), immune effector cell-associated neurotoxicity syndrome (ICANS), and prolonged cytopenias were not observed in this patient. She had complete depletion of circulating B cells that was maintained for more than 44 days. Her double-stranded DNA (dsDNA) autoantibodies and complement levels normalized. Clinically, she had improvement in proteinuria and her SLEDAI score improved from 18 to 0 post treatment [59].

Five young adult patients with refractory lupus were enrolled in a compassionate use trial of autologous CD19 CAR T cell therapy after previously failing management with conventional immunosuppressives [60]. The patients, aged 18 to 24 years, had disease durations ranging from 1 to 9 years and high baseline lupus disease activity as measured by the SLEDAI (scores 8 to 16). All had LN (stages III, IV, or III/V). In brief, the patients received lymphodepleting chemotherapy with fludarabine and CYC from day − 5 to day − 3 followed by 1 × 10^6^ CAR T cells/kg. All five patients had an in vivo expansion of the infused CD19 CAR T cells and rapid and sustained depletion of circulating B cells. All patients achieved control of LN and SLEDAI scores decreased to inactive levels (range 0–2) by 3 months. Nephritis resolved in all five of the patients, as demonstrated by normalization of proteinuria. Other manifestations including arthritis, fatigue, and fibrosis of cardiac and pulmonary valves resolved after the administration of CD19 CAR T cells. Complement C3 levels normalized, and anti-dsDNA and ANA levels significantly declined in all patients. The patients were able to discontinue both corticosteroids and hydroxychloroquine and demonstrated a durable mean remission of 9.8 months (5–17 months). Severe CRS was not observed in any of the patients, although 3 patients developed grade 1 CRS (fever alone) lasting 2–3 days that was successfully treated by administration of methimazole in four of the patients and a single infusion of tocilizumab (8 mg/kg) in one patient. No patients had signs of ICANS, or infections reported during the short-term period of the study (5–17 months). No SLE flare has been reported in any of the patients thus far despite reconstitution of patients peripheral B cells [61]. More recently, long-term follow up data up to 29 months post infusion demonstrated a lasting remission in all patients without any immunosuppressive medications [62].

Additionally, a 32 year old woman with SLE was successfully treated with CD19-targeted CAR T cells [63]. Lupus was diagnosed during pregnancy. Clinical manifestations included serositis, nephritis, cytopenias, immunologic findings (ANA, dsDNA, hypocomplementemia), and concern for central nervous system involvement. She remained refractory to treatment with multiple agents including tacrolimus, belimumab, CYC, and rituximab. Prior to undergoing conditioning treatment for cell retrieval, she was weaned off immunosuppression three weeks prior to leukapheresis except for glucocorticoids, which were tapered to 5 mg daily. She tolerated CAR T cell treatment with no reported adverse effects, and after three months she achieved lupus low disease activity per low lupus disease activity state (LLDAS) criteria, proteinuria normalized, and she no longer had detectable circulating anti-dsDNA antibodies three months after treatment. Of note, B cells returned to the peripheral blood within 2 months without associated disease recurrence.

Importantly, the feasibility of manufacturing autologous CAR T cells from patients with lupus has been demonstrated to be achievable [64]. Manufacturing failures can occur in patients with active malignancy who are heavily immunosuppressed and are often cytopenic. Similar concerns exist in patients with SLE. All SLE patients studied were on glucocorticoids and received a wide array of immunosuppressive treatment prior to cell collection for CAR T generation. Glucocorticoids were tapered to a maximum of 10 mg/day one week prior to apheresis while other immunosuppressive agents were held two weeks beforehand. Despite patients being on low dose glucocorticoids, enough autologous T cells were successfully collected to generate ten times the minimum amount of CAR T cells needed for a single clinical infusion [64].

CAR T cells targeting the B cell Maturation Antigen (BCMA) receptor are FDA approved in patients with multiple myeloma and have a similar safety profile to CD19 CAR T cells. One strategy is to deplete both pathogenic B cells and antibody producing plasma cells by targeting both BCMA and CD19. To this end, Zhang et al. developed a CD19-BCMA compound CAR T cell product that has been successfully to eliminate donor specific alloantibodies prior to stem cell transplantation [65]. The use of the compound CD19-BCMA CAR T cells in a patient with SLE and stage IV diffuse large B cell lymphoma was reported [66]. She received fludarabine and CYC lymphodepleting chemotherapy followed by an infusion of 5.3 × 10^6^ CD19-BCMA CAR T cells/kg. B cell depletion was rapid and complete through 198 days after treatment. The patient was able to discontinue glucocorticoid therapy and achieved normal complement levels and undetectable ANA levels after treatment. She remained in remission 23 months after treatment despite B cell reconstitution, but specific immunophenotyping of the reconstituted B cells was not reported [66]. Safety and preliminary efficacy of a combination of CD19 and BCMA CAR T cells in SLE has also been reported [67]. 12 patients with SLE refractory to multiple lines of immunosuppressive therapies including rituximab and belimumab were enrolled in a clinical trial. Patients received 1 or 2 × 10^6 of each CD19 and BCMA CAR T cells. The infusions were safe with only mild grade 1 CRS and no neurologic toxicity. Anemia was reported in all (Table 1). Mild infections were reported in 4 patients within 6 months of infusion [67]. After infusion of CD19/BCMA CAR T cells all patients met LLDAS criteria and were able to discontinue all immunosuppression including glucocorticoids. B cell aplasia was noted for approximately 3 months after CAR T cell therapy and no SLE flares have occurred at a median follow up of 118.5 (45–524) days [67].

**Table 1:** A review of CAR T therapy for humans patients with SLE:

Author	Number of patients	Duration of disease (mean)	Disease manifestations	LN class	Previous treatment	Follow up	Side effects
Mougiakakos	1	Approximately 2 years	LN, pericarditis, pleurisy, rash, arthritis, and a history of Libman–Sacks endocarditis	Class IIIa	Hydroxychloroquine, High-dose glucocorticoids, CYC, MMF, Tacrolimus, Belimumab, and Rituximab	44 days after infusion reported	None reported
Zhang	1	20 years	SLE features not reported; also had stage IV diffuse large B cell lymphoma	N/A	Prior SLE treatment not reported; R-CHOP for DLBCL	37 weeks	None reported
Mackensen	5	1–9 years (4.6)	LN, carditis, lung disease, and arthritis. No CNS disease.	Class III, IV, or III/V in all 5 patients	Pulsed glucocorticoids (5/5), hydroxychloroquine (5/5),MMF (5/5), belimumab (5/5), azathioprine (2/5), and CYC (3/5)	5–17 months, mean 9.8 months	CRS grade 1 (fever) in 3 patients
Taubmann	1	Less than one year	Lupus nephritis, serositis, and concern for cerebritis	Class IV	Tacrolimus, Belimumab, CYC, and Rituximab	More than 150 days	None reported
Zhang	12	Not reported	Not reported	Not reported	Rituximab and Belimumab	45–524 days	Grade 4 hematologic toxicity (11/12); Grade 3 hematologic toxicity (1/2) 2 pts with neocoronavirus; 1 patient with GI infection; 1 patient with pulmonary infection

## Discussion and conclusions

In conclusion, despite recent expansion of available treatments for SLE, remission rates remain unacceptably low, and morbidity and mortality rates remain unacceptably high. This is especially true for patients with SLE onset in childhood who have worse disease severity and outcomes when compared to adult-onset SLE. B cell directed CAR T cell therapy offers significant promise for improving outcomes for refractory pediatric disease in particular. As described above, CAR T cell therapy has demonstrated safety and efficacy in treating refractory SLE in young adults with durability reported to last 2 years after receiving a single infusion. Novel therapeutics have recently been approved to treat lupus nephritis, including in the induction phase of therapy; however, most of these agents serve as add-on therapies [68]. The potential of CAR T cell therapies to reduce the need for other potentially toxic medications including glucocorticoids in children and adolescents with SLE is therefore particularly encouraging. The safety profile to date for the SLE population is more favorable compared to the oncology population due to a smaller target T cell population, and therefore a lower underlying risk of CRS.

CAR T cells enable the elimination of tissue-resident B cells that may still contribute to disease pathogenesis after treatment with the other currently available B cell depleting agents such as Rituximab and Belimumab. However, the peripheral B cell compartment most often does recover, and no significant side effects have been reported to date in this patient population. Notably, the recently reported safety signal of T cell lymphomas from CAR T is rare and has not been reported in patients without an underlying primary malignancy [69]. As limited numbers of patients have underwent CAR T therapy for autoimmune diseases, further data is required to assess its long-term safety. Controlled clinical trials of CAR T therapy in both adult and pediatric lupus will provide further insight into the efficacy, safety, and durability of this therapy (Table 2).

**Table 2:** A review of the current clinical trials of CAR T for SLE

Clinical Trial:	Location:	Eligibility Criteria:	Ages in years	Infusion Dose:	Disease Activity Requirement:	Primary Outcome:
Dual Target CAR T cell Treatment for Refractory SLE Patients	Department of Rheumatology, Ren Ji Hospital South Campus, School of Medicine, Shanghai JiaoTong University Shanghai, Shanghai, China	Negative serum or urine pregnancy Negative pregnancy test at screening and baseline Subjects agree to take effective contraceptive measures during the trial until at least 1 year after CAR T cells infusion. WBC ≥ 2.5 × 10^9/L NEUT ≥ 1 × 10^9/L, BPC ≥ 50 × 10^9/L	18–70	DL-1: 0.5 ± 20%×10^5/kg, DL1: 1 ± 20%×10^5/kg, DL2: 2 ± 20%×10^5/kg DL3: 3 ± 20%×10^5/kg	SELENA-SLEDA I ≥ 8	Dose limiting toxicity
Study of Therapeutic Efficacy of Anti-CD19 CAR T cells in children with Refractory SLE	The Children’s Hospital of Zhejiang University School of Medicine	Normal cardiac, renal, and kidney function	5–18	Three dose groups (1 × 105/kg, 3 × 105/kg, 5 × 105/kg)	Still in moderate to severe disease activity despite ≥ 3 M of high dose glucocorticoids (prednisone ≥ 1 mg/kg/d or other equivalent amount of other steroids), hydroxychloroquine and at least 2 of the following treatments (CYC, MMF, azathioprine, methotrexate, cyclosporine, tacrolimus, sirolimus, leflunomide, telitacicept, beliumab, and rituximab); or Intolerant to standard treatments SLEDAI 2 K score ≥ 8 points	Safety for 3 months
A Study of CD19 Redirected Autologous T cells for CD19 Positive Systemic Lupus Erythematosus (SLE)	Shanghai Jiaotong University School of Medicine, Renji Hospital Shanghai, China	Creatinine < 1.5 mg/dl cardiac ejection fraction > 55% hemoglobin > 9 g/dL Bilirubin < 2.0 mg/dl	18–69	1E6 ~ 1E7 CD19-CAR positive T cells	Not specified	Safety of CAR T cells for 6 weeks
A Clinical Study on the Safety and Efficacy of BRL-301 (Allogeneic Chimeric Antigen Receptor T cell Injection Targeting CD19 Gene) in the Treatment of Refractory SLE	The First Affiliated Hospital, Zhejiang University School of Medicine Hangzhou, Zhejiang, China	Adequate bone marrow, liver coagulation and cardiac function Contraceptive measures or abstain from sex within at least 6 months after infusion	18–65	Not specified	At least one BILAG2004 Class A or two Class B score, or both; SELENA-SLEDAI score ≥ 8 points;	Safety of BRL-301 in SLE for 12 months
Clinical Trial for the Safety and Efficacy of CD19/BCMA Chimeric Antigen Receptor T cells Therapy for Patients With Refractory SLE	The First Affiliated Hospital, College of Medicine, Zhejiang University Hangzhou, Zhejiang, China	SLE with positive CD19/BCMA expression, and the conventional treatment is not effective and (or) no effective treatment Estimated survival time > 12 weeks; Negative urine pregnancy test before the start of administration and agreed to take effective contraceptive measures throughout the study	Child, adult, older adult	Not specified	Not specified	Safety and dose limiting toxicity
Phase I Clinical Study of GC012F Injection in Treatment of Refractory SLE	The First Affiliated Hospital, College of Medicine, Zhejiang University Hangzhou, Zhejiang, China	Hemoglobin ≥ 85 g/L; WBC ≥ 2.5 × 10^9/L; NEUT ≥ 1 × 10^9/L; PLT ≥ 50 × 10^9/LAST/ALT below 2 times the upper limit of normal; Creatinine clearance ≥ 30 mL/min; blood bilirubin ≤ 2.0 mg/dl; echocardiography indicates that the ejection fraction is ≥ 50%;	18–70	Not specified	SELENA-SLEDAI ≥ 8	Proportion of subjects with drug limiting toxicity
An Open-label, Multi-center, Phase 1/2 Study to Assess Safety, Efficacy and Cellular Kinetics of YTB323 in Participants With Severe, Refractory SLE	Novartis Investigative Sites	Adequate renal, hepatic, cardiac, hematological, and pulmonary function	18–65	Not specified	Failure to respond to two or more standard immunosuppressive therapies SLEDAI-2 K ≥ 8 (not including the SLEDAI-2 K domains of lupus headache, cerebrovascular accident, organic brain syndrome) and at least one of the following significant SLE related organ involvements: (Renal, moderate, or severe peri/myocarditis, moderate or severe pleuritis or other lung involvement, Vasculitis)	Number of participants with AEs and SAEs and long term safety follow up 1–2 years
Descartes-08 for Patients With SLE	Profound Research LLC Oceanside, California, United States	SLE at time of screening	Over 18	Not specified	Active symptoms despite recent or ongoing immunosuppressive therapy with glucocorticoids and at least 2 other immunosuppressive medications being tried for at least 12 weeks within 24 months of screening. At least one of: anti-dsDNA, anti-histone, anti-chromatin, and/or anti-Sm antibodies detectable at screening as assessed by a CLIA-certified laboratory.	Assess safety and tolerability of Descartes-08 in patients with SLE
Safety/Phase I Study of PiggyBac Transposon Mediated Chimeric Antigen Receptor T cells Targeting CD-19 in Thai Patients With Refractory SLE	King Chulalongkorn Memorial Hospital Bangkok, Please Select, Thailand	Participants of child-bearing or child-fathering potential must agree to practice birth control from enrollment until four months after receiving CAR T cell infusion	18–60	Not specified	1 Persistently active SLE requiring ongoing maintenance therapy (if not contraindicated) with: Antimalarial drug. MMF (minimum daily dose of 1500 mg) or azathioprine (minimum daily dose of 1.5 mg/kg). Patients must also need a minimum daily dose of 7.5 mg prednisolone for lower disease activity maintenance or have a SLEDAI score of 8 or higher.	Safety
A Phase 1/2 Open-label Study to Evaluate the Safety, Tolerability, Pharmacokinetics, Pharmacodynamics, and Efficacy of IMPT-514 in Participants with Active, Refractory LN and SLE	University of California, Los Angeles (UCLA) Medical Center University of California San Francisco University of Iowa Henry Ford Health System University of Cincinnati (UC)	Weight > 45 kg at enrollment; adequate blood pressure control	> 18	Not specified	Not specified	Phase I: Incidence of dose limiting toxicities (DLTs), serious adverse events (SAEs), and other treatment-emergent adverse events (
An Exploratory Clinical Study of Anti-B cell Maturation Antigen (BCMA)/Cluster of Differentiation Antigen 20(CD20) Chimeric Antigen Receptor Autologous T cell Product (C-CAR168) in the Treatment of Autoimmune Diseases Refractory to Standard Therapy	Department of Rheumatology, RenJi Hospital, School of Medicine, Shanghai JiaoTong University Shanghai, Shanghai, China	Adequate bone marrow, coagulation, cardiopulmonary, liver, and renal function.	18–70	Not specified	Remains disease active or relapses after treatment with standard of care therapy for at least 8 weeks with the dose stable for more than 2 weeks; patients should have been treated at least two immunosuppressants	Incidence of Adverse Events [Safety and Tolerability
A Phase 1/2, Open-label Study to Evaluate the Safety and Efficacy of Autologous CD19-specific Chimeric Antigen Receptor T cells (CABA-201) in Subjects with Active SLE	UC Davis Health Massachusetts General Hospital Brigham and Women’s Hospital University of Minnesota Columbia University Irving Medical Center University of Rochester UNC Chapel Hill Children’s Hospital of Philadelphia University of Texas MD Anderson Cancer Center	Positive ANA titer or anti-dsDNA antibody at screening.	18–65		For LN subjects only, active, biopsy-proven LN class III or IV, with or without the presence of class V, according to 2018 Revised International Society of Nephrology/Renal Pathology Society (ISN/RPS) criteria For non-renal SLE subjects only: Active, moderate to severe SLE	To evaluate incidence of adverse events [ Time Frame: Up to 28 days after CABA-201 infusion
BCMA-CD19 cCAR T cell Treatment of Relapsed/Refractory SLE	Zhongshan People’s Hospital Zhongshan, Guangdong, China	Expected survival period ≥ 3 months; Serum creatinine < 221.0µmol/L (2.5 mg/dl); AST/ALT below 3 times the upper limit of normal, blood bilirubin < 34.2 µmol/L (2.0 mg/dl); Cardiopulmonary function is basically normal, echocardiography indicates that the ejection fraction is > 50%, and the oxygen saturation is above 94% in the resting state without oxygen; No obvious active infection; Physical fitness score 0 ~ 2 points (ECOG standard); No pregnancy	18–65	Not specified	Not specified	The number and incidence of adverse events after BCMA-CD19 cCAR T cell infusion [ Time Frame: 3 months after CAR infusion
A Clinical Study on the Safety and Efficacy of Universal CAR T cells (BRL-301) in the Treatment of Relapse or Refractory Autoimmune Diseases	Shanghai ChangZheng hospital Shanghai, Shanghai, China	Positive expression of CD19 on peripheral blood B cells determined by flow cytometry. Bone marrow hematopoietic function needs to meet: (a) White blood cell count ≥ 3 × 10^9/L (b) Neutrophil count ≥ 1 × 10^9/L (no colony-stimulating factor treatment within 2 weeks before examination); (c) Hemoglobin ≥ 60 g/L. Liver function: ALT ≤ 3 x ULN, AST ≤ 3 x ULN, TBIL ≤ 1.5 x ULN(excluding Gilbert syndrome, total bilirubin ≤ 3.0 x ULN) (No requirements for conditions caused by the disease itself). Renal function: creatinine clearance rate (CrCl) ≥ 60 ml/minute(Cockcroft/Fault formula). Coagulation function: International standardized ratio (INR) < 1.5 x ULN, prothrombin time(PT) < 1.5 x ULN. Cardiac function: Good hemodynamic stability. Female subjects with fertility and male subjects whose partners are women of childbearing age are required to use medically approved contraception or abstinence during the study treatment period and at least 6 months after the end of the study treatment period; Female subjects of childbearing age tested negative for serum HCG within 7 days before enrollment in the study and were not in lactation.	18–65	Not specified	Not specified	Safety outcomes Time Frame: 12 months

### Electronic supplementary material

Below is the link to the electronic supplementary material.

Supplementary Material 1

## Data Availability

Not applicable.

## Abbreviations

CAR T: Chimeric antigen receptor T cells
Cyc: Cyclophosphamide
ESKD: End stage kidney disease
SLEDAI: SLE disease activity index
LLDAI: Lupus low disease activity index
SLE: Systemic lupus erythematosus
