# Supplementary material for: CAR T cell therapy for refractory pediatric systemic lupus erythematosus: a new era of hope?
Source: Pediatr Rheumatol Online J. 2024 Aug 8;22:72. doi: 10.1186/s12969-024-00990-4 (PMC11308704; doi:10.1186/s12969-024-00990-4)
Supplement: Supplementary file 1 — Supplementary Material 1 [file 12969_2024_990_MOESM1_ESM.pdf]

March 15th, 2024

To the Editor:

Attached please find our manuscript submission entitled "**CAR T cell Therapy for Refractory Pediatric Systemic Lupus Erythematosus: A New Era of Hope?**"

Our work serves to characterize and review CAR T as an emerging novel therapy systemic lupus erythematosus (SLE). It is hypothesized that by persistently eliminating both B cells and plasmablasts, CAR T therapy can halt autoimmunity and prevent organ damage in patient's refractory to current B cell-depleting treatments. We summarize the current preclinical and clinical data utilizing CAR T cells for SLE of this promising emerging therapy to date for refractory SLE and discuss the future of this treatment modality for lupus.

This manuscript is not under consideration elsewhere. This work has not been presented. All of the authors have reviewed the manuscript and associated revisions. There are no conflicts of interest.

We sincerely appreciate the time taken in consideration of this manuscript for publication in your prestigious journal. Please contact me at your convenience if there are questions regarding this submission by the email listed below. Thank you for your consideration.

Best regards,

Stojkic

Ivana Stojkic, MD
Fellow, Pediatric Rheumatology
Department of Pediatric Rheumatology, Nationwide Children's Hospital
Ivana.stojkic@nationwidchildrens.org
